# In Vitro Fermentation Patterns of Rice Bran Components by Human Gut Microbiota

**DOI:** 10.3390/nu9111237

**Published:** 2017-11-12

**Authors:** Tung Pham, Brett J. Savary, Keat (Thomas) Teoh, Ming-Hsuan Chen, Anna McClung, Sun-Ok Lee

**Affiliations:** 1 Department of Food Science, Division of Agriculture, University of Arkansas, Fayetteville, AR 72701, USA; tungphamm@gmail.com; 2 Arkansas Biosciences Institute, Arkansas State University, Jonesboro, AR 72401, USA; bsavary@astate.edu (B.J.S.); tom.teoh@gmail.com (K.(T.)T.); 3 College of Agriculture and Technology, Arkansas State University, Jonesboro, AR 72401, USA; 4 USDA Agricultural Research Service, Dale Bumpers National Rice Research Center, Stuttgart, AR 72160, USA; Ming.Chen@ars.usda.gov (M.-H.C.); anna.mcclung@ars.usda.gov (A.M.)

**Keywords:** feruloylated arabinoxylan oligosaccharides, rice bran polyphenols, short-chain fatty acids, gut microbiota, prebiotic, colon health

## Abstract

Whole grain rice is a rich source of fiber, nutrients, and phytochemicals that may promote gastrointestinal health, but such beneficial components are typically removed with the bran during polishing. Soluble feruloylated arabinoxylan oligosaccharides (FAXO) and polyphenols (RBPP) isolated from rice bran are hypothesized to have positive impacts on human gut microbiota through a prebiotic function. Using an in vitro human fecal fermentation bioassay, FAXO and RBPP treatments were assessed for short-chain fatty acids (SCFA) production patterns and by evaluating their impacts on the phylogentic composition of human gut microbiota by 16S rRNA gene sequencing. Fresh fecal samples collected from healthy adults (*n* = 10, 5 males, 5 females) were diluted with anaerobic medium. Each sample received five treatments: CTRL (no substrates), FOS (fructooligosaccharides), FAXO, RBPP, and MIX (FAXO with RBPP). Samples were incubated at 37 °C and an aliquot was withdrawn at 0, 4, 8, 12, and 24 h Results showed that SCFA production was significantly increased with FAXO and was comparable to fermentation with FOS, a well-established prebiotic. RBPP did not increase SCFA productions, and no significant differences in total SCFA production were observed between FAXO and MIX, indicating that RBPP does not modify FAXO fermentation. Changes in microbiota population were found in FAXO treatment, especially in *Bacteroides*, *Prevotella*, and *Dorea* populations, indicating that FAXO might modulate microbiota profiles. RBPP and MIX increased *Faecalibacterium*, specifically *F. prausnitzii*. Combined FAXO and RBPP fermentation increased abundance of butyrogenic bacteria, *Coprococcus* and *Roseburia*, suggesting some interactive activity. Results from this study support the potential for FAXO and RBPP from rice bran to promote colon health through a prebiotic function.

## 1. Introduction

Interactions of human gut microbiota and non-digested dietary components play important roles in health and disease [1,2]. This involves a variety of metabolic functions, including energy harvest and storage. Furthermore, the gut microbiota interacts with the host immune system, providing signals to promote the maturation of immune cells and the normal development of immune functions [3]. Factors affecting gut microbial populations includes mode of birth, age and diet [2]. In recent years, there has been a growing interest in whole grain rice and functional rice bran products [4,5] that reach the colon, such as prebiotics, dietary fiber, and other nondigested dietary components, such as polyphenolics. There they modulate beneficial gut microbiota, supporting production of short-chain fatty acids (SCFA), which have been proved to confer positive colonic health benefits [6]. Bran components and their fermentative products may also contribute to sustaining the epithelial barrier function and associated innate immunity [7,8].

A dietary prebiotic is “a substrate that is selectively utilized by host microorganisms conferring a health benefit” ([9], p. 493). Only a few non-digestible oligosaccharides are established prebiotics, including fructooligosaccharides (FOS) and galactooligosaccharides (GOS). These compounds have been reported to stimulate *Bifidobacterium* and *Lactobacillus* population, which are considered beneficial bacteria and are often targets for dietary intervention [10]. Recently, the prebiotic definition was expanded to include non-carbohydrate substances and alternative application sites [9].

The non-starch polysaccharide arabinoxylan (AX) present in cereal bran fiber, and non-digestible oligosaccharides derived from it, have been proposed to be candidate prebiotics since they appear to confer a degree of fermentation selectivity [11]. Arabinoxylan oligosaccharides isolated from cereal bran such as wheat, rye, and corn have shown to exert prebiotic-like properties in that they may pass through the upper gastrointestinal tract undigested to the colon where they are hydrolyzed and subsequently fermented by gut microbiota to produce SCFA [12,13].

Ferulic acid is a phenolic acid that is covalently bound to AX (attached by O5 ester bond to some arabinofuranosides) and can form intermolecular cross-links that immobilize AX within the insoluble fiber matrix [14,15]. Feruloylated arabinoxylan oligosaccharides (FAXO) for biofunctional studies are isolated from cereal bran by autohydrolytic [16], chemical [17], and enzymatic [18] methods. FAXO may provide increased bioavailability for ferulic acid in the colon environment, where microbial esterases can release them, providing anti-inflammatory and other effects associated with its high antioxidant potential [19,20,21,22]. Functional studies of FAXO have focused on those isolated from wheat and maize brans, while there is considerable variation in AX fine structure between cereal species and likely varieties within species [15,16,23].

Polyphenols and their derived products can also positively affect the intestinal ecology [24,25]. In vitro fermentation of grape seed flavanol fractions showed the changes of the gut microbiota composition by selectively inhibiting pathogen growth and stimulate the growth of beneficial bacteria, thus influencing the microbiota composition [26]. A randomized, controlled, double-blind, crossover intervention study with cocoa flavanols showed selective stimulation of beneficial gut microbiota in humans [27].

Feruloylated arabinoxylan and polyphenols are abundant in whole grain rice and pigmented rice bran [28,29]. However, these components are largely removed from rice during polishing [4]. Rice bran FAXO and polyphenols (RBPP) have not been studied for their potential colon health promoting properties, particularly prebiotic activities. Furthermore, there is potential for synergistic activities between FAXO and RBPP in improving colonic health. Therefore, the objectives of the present study with rice bran FAXO and RBPP were to determine fermentative SCFA production patterns by human gut microbiota and their ability to stimulate beneficial human gut microbiota populations.

## 2. Materials and Methods

### 2.1. Substrates and Standards

Feruloylated arabinoxylan oligosaccharides were isolated from multiple batches of rice bran fiber hydrolysed in hot trifluoracetic acid essentially as described by Saulnier [17], except that the majority of material was treated with 100 mM TFA for 1 h. The pooled soluble material recovered from Amberlite XAD-4 resin was analysed for ferulic acid and neutral monosaccharide contents, which provided an approximate molar ratio for Fer:Ara:Xyl:Gal:Glu of 1:1:2.2:0.2:2. Rice bran polyphenols fraction (RBPP, 50% ethanol fraction) was isolated from red rice bran (IITA 119, PI 458466) as previously described [29]. Fructooligosaccharides (FOS) were purchased from Megazyme International Ireland Ltd. (Bray Business Park, Wicklow, Ireland). All materials were tightly sealed and stored at −20 °C until use. SCFA standards including acetic acid, propionic acid, and butyric acid were of analytical grade and were purchased from Sigma-Aldrich (St. Louis, MO, USA).

### 2.2. Subjects, Dietary Records, and Fecal Sample Collection

The study protocol was approved by University of Arkansas Institutional Review Board (IRB #13-09-080). All participants were recruited from the University of Arkansas (Fayetteville, AR, USA) and surrounding area. Thirty-two volunteers (15 males and 17 females), 21–45 years of age, participated in a screening session to sign a consent form and a screening form. Only participants who were generally healthy (18.5 ≤ body mass index (BMI) < 25) with no digestive diseases, non-smokers, not currently taking any medications, and had not taken antibiotics in the last 6 months were recruited. During screening sessions, height and weight of subjects were recorded for body mass index calculation. Medical history and bowel movement habits were also recorded to confirm eligibility of participants. Ten eligible subjects (5 males and 5 females) were selected for continuing the study. No other previous studies with rice FAXO and RBPP are available in order to perform a power analysis. Each participant received a stool collection kit (Commode Specimen Collection System; Fisher Scientific, Pittsburgh, PA, USA) one or two days before the day of experiment. Subjects were instructed to deliver a tightly sealed fecal sample within one hour of defecation. Fecal samples were immediately transferred to an anaerobic chamber upon delivery to perform the experiment.

### 2.3. In Vitro Fermentation

In vitro fermentation of substrates with the fecal inocula was carried out following the method described by Yang et al. [30]. In short, 50 mg each of FAXO, RBPP, their combination (MIX-FAXO with RBPP), and FOS used as control were mixed in 10 mL of sterile fermentation medium consisting of (per liter) peptone (2 g; Fisher Scientific), yeast extract (2 g; Alfa Aesar, Ward Hill, MA, USA), bile salts (0.5 g; Oxoid, Hampshire, UK), NaHCO_3_ (2 g), NaCl (0.1 g), K_2_HPO_4_ (0.08 g), MgSO_4_·7H_2_O (0.01 g), CaCl_2_·6H_2_O (0.01 g), l-cysteine hydrochloride (0.5 g; Sigma-Aldrich, St. Louis, MO, USA), bovine hemin (50 mg; Sigma-Aldrich), Tween 80 (2 mL), vitamin K (10 μL; Sigma-Aldrich), and 0.025% (wt/vol) resazurin solution (4 mL). Fecal slurry was prepared by vortexing 1.0 g of fecal sample with 10 mL of sterile phosphate-buffered saline until fully suspended then filtering through four layers of cotton gauge. Test tubes containing fermentation medium and treatments were then inoculated with 0.2 mL of fecal slurry. All steps for fermentation were conducted in an anaerobic chamber (Coy Laboratory Products Inc., Grass Lake, MI, USA). Test tubes were then capped, tightly sealed, and vortexed for 5 seconds to mix. Subsequently, test tubes were transferred to the incubator set at 37 °C. Immediately before incubation, 1.5 mL of the mixture was taken out from each test tube using a sterile syringe for time point 0 h into a 2-mL centrifuge tube containing 0.1 mL of 2 M KOH stop solution. Subsequent aliquots were obtained in the same manner at 4, 8, 12, and 24 h and stored at −80 °C until analysis.

### 2.4. Short-Chain Fatty Acid Analysis

Fermentation samples were thawed at room temperature and mixed with a vortex mixer. An amount of 225 µL was withdrawn from each aliquot and was combined with 25 µL of a mixture containing 5% meta-phosphoric acid and 5% copper sulfate with 50 mM 4-methyl-valeric acid added as an internal standard. After 10 min of reaction time, the mixture was centrifuged for 2 min at 12,000× *g*. A supernatant of 200 µL was transferred into a labelled tube and stored at −20 °C until analysis.

SCFA standards were prepared using 1:2 serial dilution with the stock solution containing 10% *v*/*v* of each SCFA (acetic acid, propionic acid, and butyric acid) in Milli-Q water. SCFA standards were also treated with the same mixture containing meta-phosphoric acid, copper sulfate, and 4-methyl-valeric acid as with fermentation samples.

SCFA contents in samples were measured quantitatively using a gas chromatograph with flame ionization detection (Shimadzu Corp., Kyoto, Japan) equipped with a BP21 fused silica capillary column (SGE Analytical Science/Trajan, Pflugerville, TX, USA; 30 mm × 0.25 mm, 25 µm). Temperature ramp was as following: 4 °C/min from 100 °C (2 min) to 120 °C (1 min), then 3 °C/min until 150 °C. In addition, 1 µL of treated sample (thawed and homogenized) was injected in split mode (30:1). Nitrogen was used as a carrier gas. Data were recorded and processed using the integrated Shimadzu database. Concentrations of acetic acid, propionic acid, and butyric acid were determined using a standard curve of each SCFA.

### 2.5. DNA Extraction and Sequence Analysis

Bacterial proliferation capability of each treatment was assessed by DNA sequencing analysis of samples at time point 24 h. Bacterial DNA was extracted from sample aliquots using QIAamp Fast DNA Stool Mini Kit (Qiagen, Gaithersburg, MD, USA). DNA concentrations were measured using NanoDrop 1000 (Thermo Fisher Scientific, Waltham, WA, USA). All samples were diluted with DNase- and RNase-free water to achieve concentrations of 10 ng/µL. DNA samples were then mixed with AccuPrime Pfx SuperMix (Thermo Fisher Scientific, Waltham, WA, USA) and primers and were amplified via polymerase chain reaction (PCR) using Eppendorf Mastercycler pro S (Eppendorf, Hamburg, Germany). Amplification of DNA samples were confirmed by agarose gel electrophoresis. Amplified DNA samples were normalized using SequalPrep Normalization Plate Kit (Thermo Fisher Scientific, Waltham, WA, USA) before pooling to make DNA sample library. Sequencing based on 16S-rRNA V4 region was performed using an Illumina MiSeq platform (Illumina, San Diego, CA, USA) with the method developed by Kozich et al. [31]. Raw sequencing data acquired from Illumina BaseSpace were processed with a bioinformatics tool QIIME (Quantitative Insights into Microbial Ecology) pipeline (version 1.9.0) [32].

### 2.6. Statistical Analyses

All statistical analyses were carried out by JMP software (version 12; SAS Institute, Cary, NC, USA), using one-way ANOVA for comparing three or more data sets or paired t-test for comparing two data sets. A Tukey test was performed to correct for multiple comparisons. Data are presented as Mean ± SEM (Standard Error of Mean) unless specified as standard deviation (SD). Statistically significance was accepted at *p* < 0.05.

## 3. Results

### 3.1. Subject Characteristics

In the present study, 10 subjects (5 males, 5 females) were recruited. Participant information including age, height, weight, and body mass index (BMI) is shown in Table 1. BMI of all subjects were within normal range (19.6–24.6).

### 3.2. Short-Chain Fatty Acid Analysis

No significant differences in total and individual SCFA production were observed between males and females. SCFA concentrations were measured at time points from 0 h to 24 h (Table 2). FAXO appeared to be a preferred substrate by the microbiota as evidenced by the increase in total SCFA. Compared to FOS, a widely-recognized prebiotic, total SCFA production of FAXO was very comparable at later time points of 12 h and 24 h as no significant differences were found. However, at time point 4 h and 8 h, SCFA production of FOS was significantly higher compared to FAXO (*p* < 0.05), indicating different fermentation patterns of FOS and FAXO. FAXO appeared to have slower and steadier fermentation rates throughout the incubation period compared to FOS, which were rapidly fermented in the beginning (time point 4 h and 8 h) but then slowed down over time.

As expected for saccharolytic fermentation, RBPP was not a significant source of SCFA. Furthermore, there was no significant difference in total SCFA production between FAXO and MIX (FAXO with RBPP) at any time points, indicating that RBPP or its potential metabolic products did not affect SCFA production from FAXO.

**Figure 1:**
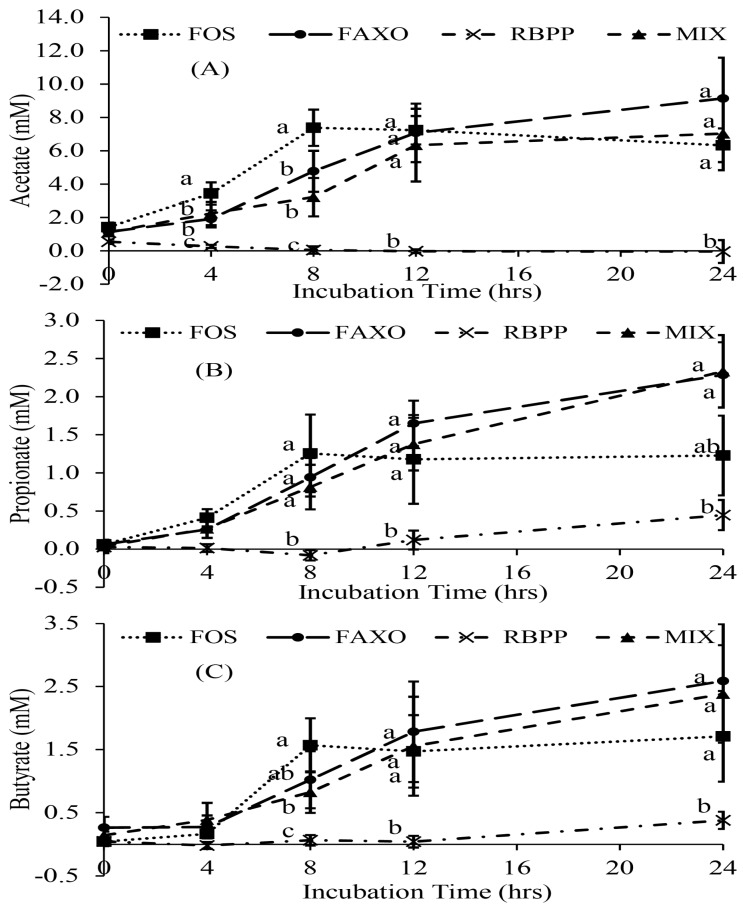
Individual SCFA (Short-Chain Fatty Acid) production during in vitro fermentation. (**A**) Acetate production; (**B**) propionate production; (**C**) butyrate production. Data are expressed as mean + SEM. Different letters at the same incubation time denote significant difference (*p* < 0.05). FOS: fructooligosaccharides, FAXO: feroloylated arabinoxylan oligosaccharides, RBPP: rice bran polyphenols, MIX: mixture of FAXO and RBPP.

Individual SCFA production (acetate, propionate, and butyrate) was also investigated (Figure 1). Acetate (Figure 1A) showed to be the predominant SCFA produced as its concentration at time point 24 h is 3–4 times higher compared to propionate and butyrate (Figure 1B,C), which were produced at similar levels. Acetate production of all treatments also exhibited similar trends as observed in total SCFA production. In particular, acetate production of FOS were significantly higher than that of FAXO at time point 4 h and 8 h but not at time point 12 h and 24 h. However, for propionate and butyrate production, no significant differences were found at time point 4 h and 8 h when comparing FOS and FAXO. No additive effects between FAXO and RBPP were observed as there was no significant difference between FAXO and MIX at any time point for all three individual SCFA.

### 3.3. Microbiota Analysis

**Figure 2:**
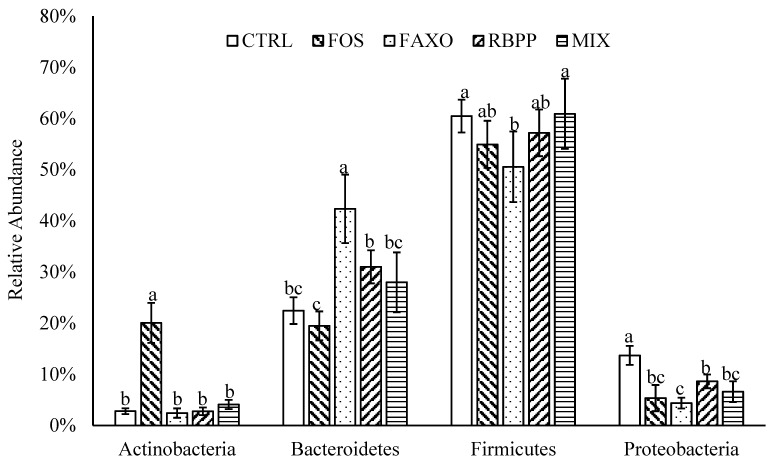
Microbiota composition at phylum level after 24 h incubation with different substrates. Data are expressed as mean ± SEM. Different letters in the same phylum denote significant difference (*p* < 0.05). CTRL: no substrate, FOS: fructooligosaccharides, FAXO: feroloylated arabinoxylan oligosaccharides, RBPP: rice bran polyphenols, MIX: mixture of FAXO and RBPP.

The 16S rRNA sequencing data were analyzed to investigate the changes in microbiota composition after 24 h incubation with FAXO, RBPP, and MIX compared with CTRL (control, no substrate) and FOS. At phylum level, four major phyla were identified in all samples including Actinobacteria, Bacteroidetes, Firmicutes, and Proteobacteria (Figure 2). After 24 h of incubation, different substrates appeared to be able to modulate the microbiota composition significantly. FAXO appeared to increase Bacteroidetes and decrease Firmicutes abundance significantly compared to CTRL (*p* < 0.05). Proteobacteria population was also suppressed significantly in FAXO compared to CTRL (*p* < 0.05). RBPP alone and in combination with FAXO (MIX) only affected the Proteobacteria population as evidenced by a significant decrease in the abundance of this phylum compared to CTRL (*p* < 0.05). However, RBPP seemed to decrease Proteobacteria to a lesser extent compared to FAXO. On the other hand, FOS, which is used as a positive control, showed a significant increase in Actinobacteria population and a significant decrease in Proteobacteria compared to CTRL (*p* < 0.05). However, no significant changes were found in the abundance of Bacteroidetes and Firmicutes with FOS treatment compared to CTRL.

**Figure 3:**
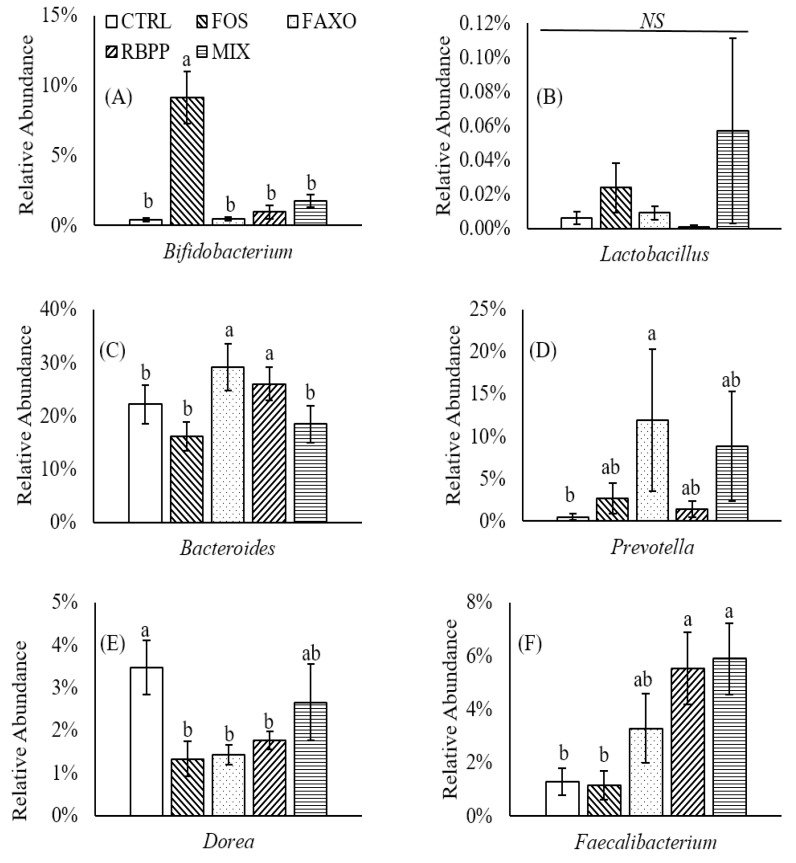
Change in abundance of different genera after 24 h incubation with different substrates. (**A**) *Bifidobacterium*; (**B**) *Lactobacillus*; (**C**) *Bacteroides;* (**D**) *Prevotella*; (**E**) *Dorea*; (**F**) *Faecalibacterium*. Data are expressed as mean ± SEM. Different letters denote significant difference (*p* < 0.05). CTRL: no substrate, FOS: fructooligosaccharides, FAXO: feroloylated arabinoxylan oligosaccharides, RBPP: rice bran polyphenols, MIX: mixture of FAXO and RBPP.

Relative abundance of representative genera was also investigated. Results showed that FOS increased *Bifidobacterium* abundance dramatically, which indicated FOS were utilized by *Bifidobacterium* sp. (*p* < 0.05) (Figure 3A). An increase in *Lactobacillus* was observed; however, it was not significant compared to CTRL (Figure 3B). No significant changes were observed in *Bifidobacterium* and *Lactobacillus* with FAXO treatment. Similarly, RBPP and MIX did not appear to significantly affect the abundance of *Bifidobacterium* and *Lactobacillus*. Overall, FAXO, RBPP, and MIX did not seem to exert any effects on *Bifidobacterium* and *Lactobacillus*, two genera that are often targets for prebiotic action as they are commonly associated with many health benefits. However, considering other genera, significant differences were observed. Specifically, *Bacteroides* was increased significantly in FAXO and RBPP compared to both CTRL and FOS (*p* < 0.05) (Figure 3C). MIX treatment, however, did not affect *Bacteroides* abundance. FAXO also appeared to increase *Prevotella* abundance significantly compared to CTRL (*p* < 0.05) (Figure 3D). Population of *Dorea* was also affected by FOS, FAXO, and RBPP with significant decreases compared to CTRL (*p* < 0.05) (Figure 3E). In MIX, no significant difference in *Dorea* were found compared to CTRL. *Akkermansia*, a mucus-degrading genus that has gained significant attention in recent years because of its correlation to gut health, was also detected. However, no significant differences in *Akkermansia* population were found between treatments. The average abundance of *Akkermansia* was between 0.20% and 0.41% for all treatments. *Faecalibacterium*, a butyrate-producing genus, was increased significantly in abundance with RBPP and MIX compared to CTRL and FOS (*p* < 0.05) (Figure 3F). FAXO also seemed to increase *Faecalibacterium*, but no significant difference was observed. Taken together, these results suggested that, although FAXO and RBPP did not alter *Bifidobacterium* and *Lactobacillus*, they appeared to be able to modulate other gut bacteria populations that could also play important roles in host health.

### 3.4. Relationship of Human Gut Microbiota and SCFA Production

**Figure 4:**
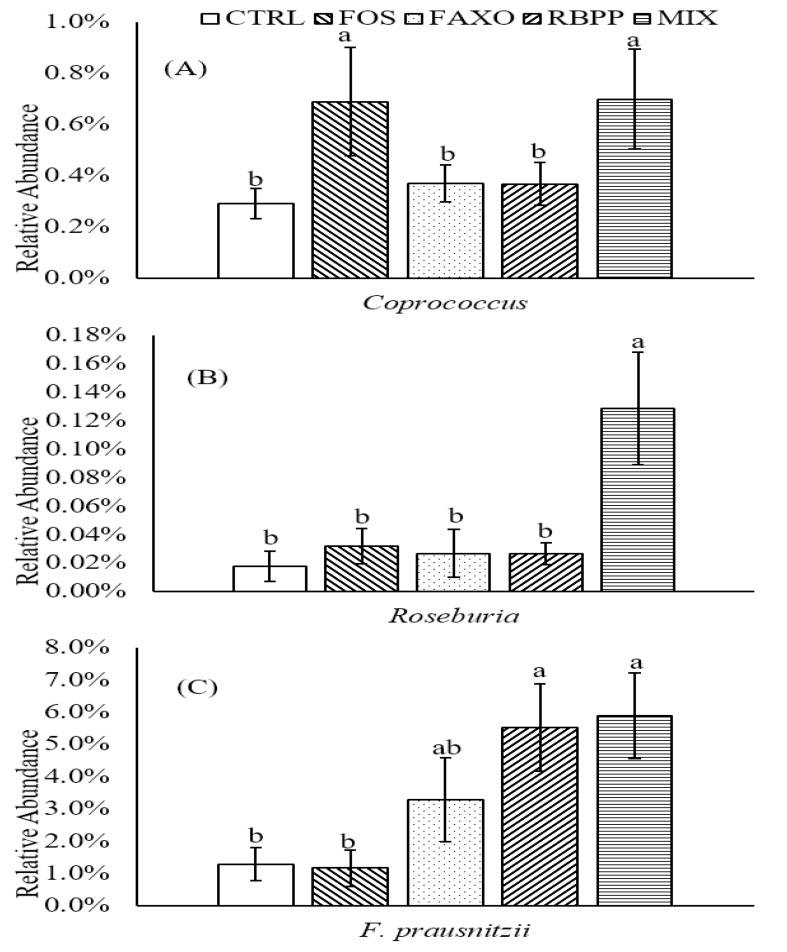
Changes in abundance of different butyrogenic bacteria after 24 h incubation with different substrates. (**A**) *Coprococcus*; (**B**) *Roseburia*; (**C**) *F. prausnitzii*. Data are expressed as mean ± SEM. Different letters denote significant difference (*p* < 0.05). CTRL: no substrate, FOS: fructooligosaccharides, FAXO: feroloylated arabinoxylan oligosaccharides, RBPP: rice bran polyphenols, MIX: mixture of FAXO and RBPP.

The production of butyrate has received much attention for its anti-inflammatory and anti-neoplastic effects on colonocytes [33,34,35]. Butyrate is not only produced directly from carbohydrate sources by butyrate-producing bacteria, but it can also be produced from acetate. As reported by Duncan et al. [36], butyrate could be converted from acetate by butyrogenic bacteria including *Coprococcus sp.*, *Roseburia sp.*, and *Faecalibacterium prausnitzii*. The abundance of these bacteria groups was also assessed to evaluate the relationship of gut microbiota and SCFA production (Figure 4). Combined effects by FAXO and RBPP were observed in *Coprococcus* and *Roseburia* as evidenced by a significant increase in MIX compared to CTRL in these two genera (*p* < 0.05) while both FAXO and RBPP did not differ from CTRL (Figure 4A,B). *F. prausnitzii*, the most abundant species in *Faecalibacterium* genus, also exhibited similar trends as in its genus shown above. Comparing to CTRL, both RBPP and MIX appeared to increase the abundance of this particular species significantly (*p* < 0.05) (Figure 4C).

## 4. Discussion

In recent years, cereal bran AX and FAXO derived from them, have gained considerable interest in nutrition science for their prebiotic-like activities [37,38,39]. Metabolism of dietary prebiotics by gut microbiota results in production of SCFA and a shift in the composition colonic microbiota that is associated with improved health.

In the present study, FAXO isolated from rice bran were shown to be fermented by gut microbiota to produce SCFA. The fermentation patterns of FAXO were characterized by slower rates at time point 4 h and 8 h and faster rates at later time points compared to FOS. Rumpagaporn et al. [39] also reported similar findings in fermentation patterns of cereal arabinoxylans isolated from wheat, corn, sorghum, and rice compared to FOS (a positive control). As described in their study, fecal samples were collected from three healthy subjects and pooled. An amount of 50 mg of each substrate and 1 mL of pooled fecal slurry were used for the experiment. Arabinoxylans from different bran sources including corn and sorghum and hydrolyzed arabinoxylan products including corn, wheat, and rice were used in that study.

Total SCFA production in hydrolyzed rice bran arabinoxylan treatment were shown to have significantly higher levels compared to FOS at time point 24 h. In the present study, although total SCFA of FAXO was higher than that of FOS, no significant differences were found at time point 24 h when comparing FAXO and FOS.

Acetate was the major SCFA produced during fermentation of rice FAXO. Similar findings were also reported in wheat arabinoxylans [40], arabinoxylan oligosaccharides from brewery spent grain [41] and hydrolyzed rice arabinoxylans [39]. Acetate production of FAXO and FOS also showed similar patterns as in total SCFA production in this study. In colonic fermentation, acetate is considered the primary SCFA and is often used to monitor colonic events. In the colon, unlike propionate and butyrate, acetate is less metabolized and is readily absorbed. The presence of acetate also decreases colonic pH, which results in increased bio-availability of calcium and magnesium and inhibition of pathogenic bacteria [42,43]. In addition, acetate can be converted to butyrate. Two mechanisms that have been reported for the production of butyrate in the colon are acetate utilization and lactate fermentation [44].

The propionate production of FAXO was also comparable to FOS in this study. Other studies have shown that arabinoxylans produced relatively high propionate [38,39]. Rumpagaporn et al. [39] also reported that propionate concentration in rice arabinoxylans was significantly higher compared to FOS at time point 24 h. In this study, propionate production of FAXO at 24 h tended to be higher compared to FOS; however, the differences were not significant. After produced by gut bacteria, propionate is absorbed into bloodstream and transported to liver [43]. Propionic acid production has been shown to have beneficial health effects including lowering glucose-induced insulin secretion in isolated pancreatic islet cells of rats [45] and anti-proliferative effects on liver cancer cells [46].

Among all SCFA, butyrate has been of greatest interest due to its protective effects of colonocytes against cancer [34,35]. The combination of butyrate and mevastatin, an inhibitor of 3-hydroxy-3-methylglutaryl-coenzyme A (HMG-CoA) reductase, synergistically inhibited growth of colon cancer cells [47]. FOS has been known for the ability to increase butyrate production, hence the butyrogenic effects [48,49,50]. Fermentation of arabinoxylans generated lesser butyrate compared to FOS in a study by Rumpagaporn et al. [39]. However, in the present study, no significant differences were found between FAXO and FOS. Butyrate was the preferred energy source for colonocytes and inhibited the growth of colonic carcinoma cells [51]. As discussed above, the production of butyrate also comes from the conversion of acetate. Therefore, the production of each individual SCFA depends on other SCFA and SCFA concentrations in anaerobic fermentation can be changed interdependently.

Besides SCFA production upon fermentation, another criterion that a prebiotic must meet is the ability to selectively stimulate the growth and/or activity of intestinal bacteria associated with health and well-being. *Bifidobacteria* and *lactobacilli* were thought to be the targets of prebiotic effects since they have been the focus of research, especially *bifidobacteria* as they are more abundant in human gut microbiota than *lactobacilli*. However, in recent years, many studies have reported the potential health effects of different groups of bacteria other than *bifidobacteria* and *lactobacilli*.

FAXO appeared to be a relatively selective substrate as demonstrated in several studies. Pure cultures of different *Bifidobacterium*, *Bacteroides*, and *Lactobacillus* species were shown to efficiently utilize FAXO [52,53,54]. Another study conducted by Vardakou et al. [55] found that in vitro fermentation of arabinoxylan oligosaccharides significantly raised *Bifidobacterium* and reduced *Bacteroides* levels. In the present study, FAXO did not stimulate the growth of *Bifidobacterium* and *Lactobacillus* genus. However, when assessing populations of other bacteria genera, FAXO might be able confer certain positive health effects by modulating other genera. Specifically, FAXO increased *Bacteroides* and *Prevotella* abundance, while reducing *Dorea* abundance.

*Bacteroides* is one of the most abundant genera in the gut microbiota. Aside from its correlation with increased propionate production, *Bacteroides* has also been shown to have protective effects against the invasion of exogenous bacteria in the colon by producing antagonic substances including bacteriocins [56]. This bacterial characteristic might play an important role in establishing and maintaining the intestinal ecosystem.

It was also interesting to see an increase in *Prevotella* sp. with FAXO. This genera is often associated with people with diets high in carbohydrates and fiber [57]. However, the variation between subjects in FAXO is quite large and comparing to FOS, FAXO did not differ significantly (Figure 3D). A study conducted by De Filippo et al. [58] revealed the differences in changes in gut microbiota of European and African children when solid food was introduced. European children’s diet was rich in fat and low in fiber while African children’s diet was rich in fiber and low in fat and animal proteins. During the breast-milk feeding period, no significant differences in gut microbiota were found between two groups of children. However, when solid food was introduced, differences in bacteria populations were observed. There was a significant enrichment of *Prevotella* genus in gut microbiota of African children compared to that of European counterparts. The differences were explained by the ability to produce cellulases and xylanases of this genus. Therefore, an increase in *Prevotella* with FAXO was expected.

Compared to other genera, *Dorea* was far less studied. However, correlation between *Dorea* population and disease has been demonstrated. Specifically, it has been shown that irritable bowel syndrome (IBS) was characterized by an increase in *Dorea* population and a decrease in *Bifidobacterium* and *Faecalibacterium* [59,60]. These findings suggest that there might be a link between gut microbiota and IBS, which could potentially be used for therapeutic treatments.

As discussed above, some bacteria are also capable of converting acetate to butyrate, namely *Coprococcus*, *Roseburia*, *R. intestinalis*, and *Faecalibacterium prausnitzii* [32]. In the present study, RBPP and MIX increased the abundance of a butyrate producing genus, *Faecalibacterium prausnitzii*. In addition, it is interesting that, while both FAXO and RBPP did not appear to modulate *Coprococcus* and *Roseburia* abundance, MIX treatment showed significant increases in both genera, especially in *Roseburia*, where FOS also did not affect its population. These results suggested that there might be synergistic effects between FAXO and RBPP in modulating these gut microbiota. Although these butyrogenic bacteria were increased in abundance, no significant differences were observed in neither acetate production nor butyrate production between FAXO and MIX.

The study was conducted using an in vitro anaerobic fermentation model, which is commonly used for first assessment of impacts of various compounds on metabolic activities of gut microbiota. However, this method certainly has its limitations. First of all, since it is a closed system, metabolites produced are constrained by amounts of substrates used. Second, the end-products accumulated during the fermentation period could alter the conditions of fermentation environment and affect the formation of certain metabolites. Third, in vitro methods do not fully replicate in vivo intestinal conditions, which affects the in vivo relevance of the study. Moreover, due to limited availability of the FAXO substrate, only 10 subjects were recruited for the study. The small sample size could reduce the statistical power and undermine the treatment effects.

The present study investigated the fermentibility of FAXO and RBPP and the changes in gut microbiota during fermentation with these components. The results demonstrated that FAXO had different fermentation patterns compared to FOS. FAXO were also found to be able to modulate several bacteria populations that could contribute to overall host health.

## 5. Conclusions

Total SCFA produced in vitro from rice bran FAXO by gut microbiota fermentation was comparable with the established prebiotic FOS, while the overall fermentation pattern from FAXO was different. Furthermore, FAXO showed distinctive modulating effects on microbiota phylogenetic composition profiles. A stimulatory effect was also observed for mixed FAXO and RBPP in modulating certain butyrate-producing bacteria. This study warrants further investigation of these rice bran components to confirm the prebiotic-like properties and any cooperative activity for modulating the gut microbiota.

## Figures and Tables

**Table 1:** Subject characteristics.

Measurements	All (*n* = 10)	Male (*n* = 5)	Female (*n* = 5)
Age (year)	25.8 ± 4.7	26.0 ± 5.1	25.6 ± 4.2
Height (m)	1.7 ± 0.1	1.7 ± 0.1	1.7 ± 0.0
Weight (kg)	66.6 ± 7.3	69.8 ± 7.5	63.5 ± 5.8
Body mass index (kg/m^2^)	22.8 ± 2.0	23.1 ± 1.8	22.5 ± 2.3

Values are expressed as mean ± SD (standard deviation).

**Table 2:** Total production of SCFA (Short-Chain Fatty Acid) during in vitro fermentation with human fecal samples.

Time Point (h)	Total SCFA (mM)			
	FOS	FAXO	RBPP	MIX
0	1.5 ± 0.3 ^a^	1.5 ± 0.3 ^a^	0.6 ± 0.2 ^a^	1.3 ± 0.3 ^a^
4	4.0 ± 0.8 ^a^	2.4 ± 0.7 ^b^	0.3 ± 0.1 ^c^	2.9 ± 1.0 ^ab^
8	10.2 ± 1.1 ^a^	6.7 ± 1.8 ^b^	0.0 ± 0.2 ^c^	4.9 ± 1.7 ^b^
12	9.9 ± 1.1 ^a^	10.5 ± 2.7 ^a^	0.1 ± 0.2 ^b^	9.3 ± 3.1 ^a^
24	9.3 ± 1.1 ^a^	14.0 ± 3.6 ^a^	0.8 ± 0.7 ^b^	11.7 ± 3.2 ^a^

Values are expressed as mean ± SEM. Treatments with different superscripts within the same row are significantly different (*p* < 0.05). FOS: fructooligosaccharides, FAXO: feroloylated arabinoxylan oligosaccharides, RBPP: rice bran polyphenols, MIX: mixture of FAXO and RBPP.
